# Dengue in Malaysia: Factors Associated with Dengue Mortality from a National Registry

**DOI:** 10.1371/journal.pone.0157631

**Published:** 2016-06-23

**Authors:** Su May Liew, Ee Ming Khoo, Bee Kiau Ho, Yew Kong Lee, Mimi Omar, Vickneswari Ayadurai, Fazlina Mohamed Yusoff, Zailiza Suli, Rose Nani Mudin, Pik Pin Goh, Karuthan Chinna

**Affiliations:** 1 Department of Primary Care Medicine, Faculty of Medicine, University of Malaya, 50603 Kuala Lumpur, Malaysia; 2 Klinik Kesihatan Bandar Botanik, Ministry of Health Malaysia, 42000 Klang, Malaysia; 3 Klinik Kesihatan Kelana Jaya, Ministry of Health Malaysia, 47301 Petaling Jaya, Malaysia; 4 Klinik Kesihatan Taman Medan, Ministry of Health Malaysia, 46000 Petaling Jaya, Malaysia; 5 Klinik Kesihatan Seksyen 19 Shah Alam, Ministry of Health Malaysia, 40300 Shah Alam, Malaysia; 6 Vector-Borne Disease Sector, Ministry of Health Malaysia, 62590 Putrajaya, Malaysia; 7 National Clinical Research Center, Ministry of Health Malaysia, 50586 Kuala Lumpur, Malaysia; 8 Department of Social and Preventive Medicine, Faculty of Medicine, University of Malaya, 50603 Kuala Lumpur, Malaysia; Public Health England, UNITED KINGDOM

## Abstract

**Background:**

The increasing incidence and geographical distribution of dengue has had significant impact on global healthcare services and resources. This study aimed to determine the factors associated with dengue-related mortality in a cohort of Malaysian patients.

**Methods:**

This was a retrospective cohort study of patients in the Malaysian National Dengue Registry of 2013. The outcome measure was dengue-related mortality. Associations between sociodemographic and clinical variables with the outcome were analysed using multivariate analysis.

**Results:**

There were 43 347 cases of which 13081 were serologically confirmed. The mean age was 30.0 years (SD 15.7); 60.2% were male. The incidence of dengue increased towards the later part of the calendar year. There were 92 probable dengue mortalities, of which 41 were serologically confirmed. Multivariate analysis in those with positive serology showed that increasing age (OR 1.03; CI:1.01–1.05), persistent vomiting (OR 13.34; CI: 1.92–92.95), bleeding (OR 5.84; CI 2.17–15.70) and severe plasma leakage (OR 66.68; CI: 9.13–487.23) were associated with mortality. Factors associated with probable dengue mortality were increasing age (OR 1.04; CI:1.03–1.06), female gender (OR 1.53; CI:1.01–2.33), nausea and/or vomiting (OR 1.80; CI:1.17–2.77), bleeding (OR 3.01; CI:1.29–7.04), lethargy and/or restlessness (OR 5.97; CI:2.26–15.78), severe plasma leakage (OR 14.72; CI:1.54–140.70), and shock (OR 1805.37; CI:125.44–25982.98), in the overall study population.

**Conclusions:**

Older persons and those with persistent vomiting, bleeding or severe plasma leakage, which were associated with mortality, at notification should be monitored closely and referred early if indicated. Doctors and primary care practitioners need to detect patients with dengue early before they develop these severe signs and symptoms.

## Introduction

Dengue is the most common and serious arthropod-borne viral disease. First reported in 1779 by David Bylon during an epidemic in Indonesia, there has been a dramatic expansion in disease distribution in the last 50 years [1]. Before 1970, only nine countries had dengue epidemics. Currently, dengue is endemic in more than a hundred countries in five out of the six WHO regions [2]. It is estimated that there are 2.5 billion people living in dengue-endemic countries [3, 4].

Nearly 75% of the global dengue disease burden is in the WHO South East Asia and Western Pacific regions [3, 4]. Dengue was first reported in 1902 in Penang, Malaysia [5]. The incidence rate of dengue in Malaysia had quadrupled from 44.3 cases/100 000 in 1999 to 181 cases/100 000 in 2007 [6] and the number of reported dengue cases has increased 6.5 fold in the last decade [7]. Since 2001, the fatality rate has been 2 to 3 in a thousand cases except for 2007 where it increased to 6 in a thousand [6, 7]. The high dengue incidence, and its possible complications and fatalities, has posed a huge burden on the national health care system. During dengue epidemics, vital resources including time, hospital beds, finances and personnel are diverted from other serious disease areas.

Dengue has a wide spectrum of clinical presentations and its clinical course can be unpredictable [8]. The WHO 1997 guidelines classified dengue into undifferentiated fever, dengue fever and dengue haemorrhagic fever (DHF). DHF was further classified into four severity grades with grade 3 and 4 defined as dengue shock syndrome (DSS) [9]. However, difficulties in applying the criteria in clinical practice have led to a revision of the classification with the disease classified as severe and non-severe dengue with or without warning signs [8].

A study in Singapore of 596 dengue cases found that female gender, lower than normal haematocrit levels, abdominal distension, vomiting and fever on admission were factors associated with severe dengue [10]. Another study on 560 dengue patients in France found that plasma leakage, severe thrombocytopenia and acute hepatitis were associated with increased mortality [11]. Abdominal pain, cough and diarrhoea were found to predict development of severe complications [11]. In Vietnam, young age and female gender were found to be associated with dengue mortality [12]. Similar findings were shown in a meta-analysis by Huy et al. [13].

The identification of variables associated with poor outcomes assists health care practitioners to focus on patients at higher risk and facilitate patient flow. Therefore, this study aimed to determine the factors associated with dengue-related mortality in a cohort of patients in the Malaysian dengue registry in 2013.

## Materials and Methods

This was a retrospective cohort study of all patients in the 2013 Malaysian national dengue (e-Dengue) registry. In Malaysia, all cases of dengue diagnosed by clinical suspicion or serological confirmation must be reported to the district health authorities using an online notification system (e-Notice). Data on socio-demographic characteristics, clinical features at notification, full blood count and disease diagnosis were sent through e-Notice. The district health authorities verified the diagnosis using the WHO 1997 criteria of acute febrile illness with two or more dengue manifestations [9]. A probable dengue case was defined as an acute febrile illness with two or more clinical manifestations (headache, retro-orbital pain, myalgia, arthralgia, rash, hemorrhagic manifestations, leukopenia) in addition to supportive serology or occurrence at the same location and time as other confirmed cases of dengue fever. A confirmed dengue case is one confirmed by laboratory criteria such as dengue virus isolation, a fourfold rise in antibody titres, virus antigen detection or virus genomic sequence detection [9]. The data was then entered into the e-Dengue registry at the district health office.

In this study, data was analysed using SPSS statistical software package (version 22.0; SPSS, Chicago, IL, USA). The outcome measure used was dengue-related mortality. Socio-demographic and clinical data were described using proportions. Continuous variables were checked for normality. Associations between variables and outcomes were first analysed using univariate analysis. Variables with a p-value of less than 0.25 were selected for inclusion into the multivariate analysis model [14]. However, variables were only considered as significant if the multivariate analysis showed that the association had a p-value of less than 0.05. Multivariate analysis was performed using the Enter method.

Names and identifiers of the patients in the registry were anonymised and kept confidential. The study was approved by the Malaysian Research Ethics Committee, NMRR-14-1275-22205.

## Results

In 2013, there were 43 347 cases of dengue entered into the registry. There were 96 deaths, of which 92 were attributed to dengue. Of the dengue deaths, 8 were children, who were less than 15 years of age. The overall dengue case fatality rate was 0.2%.

**Fig 1:**
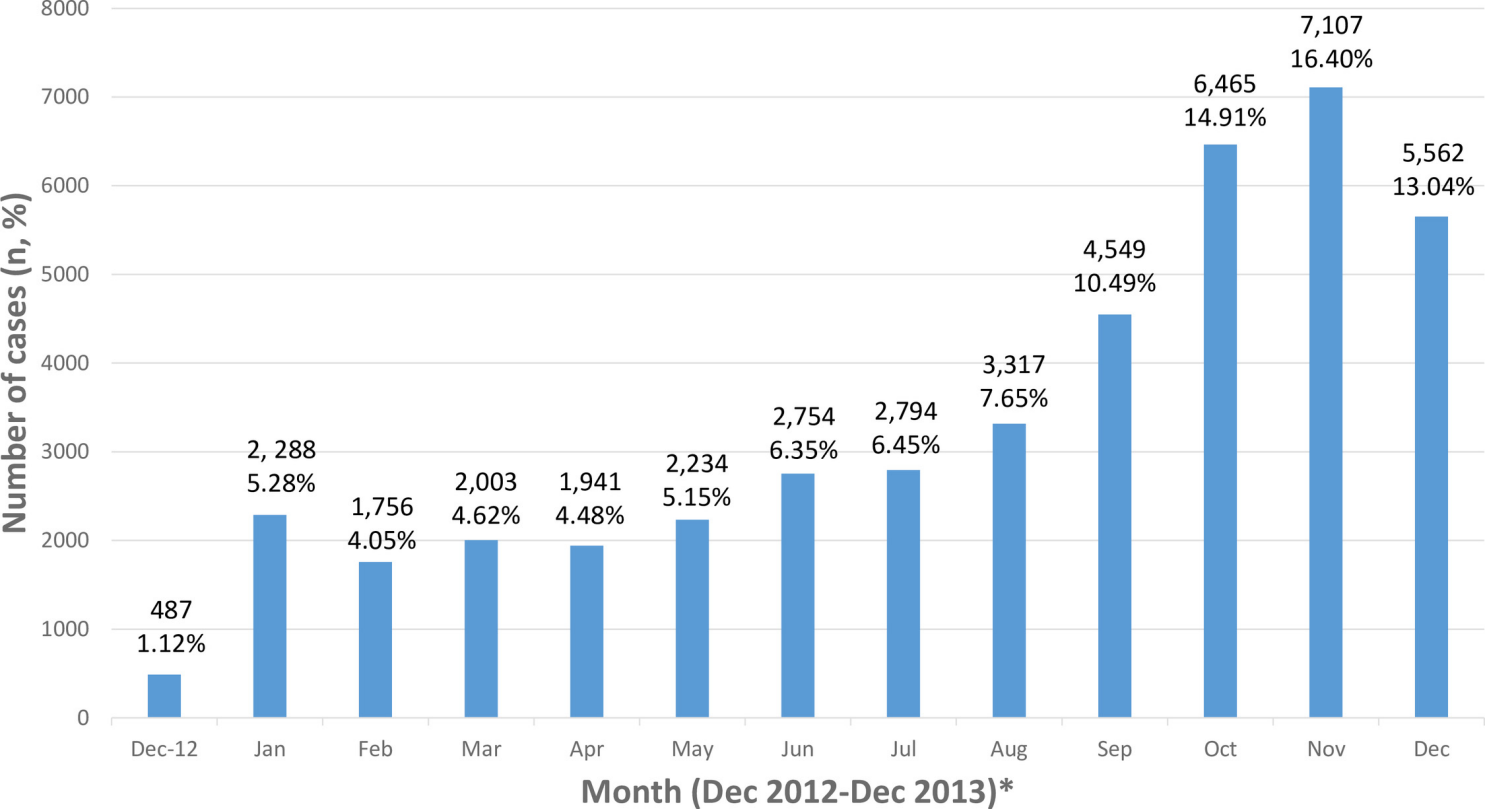
Number of dengue cases in Malaysia between December 2012-December 2013. * All months are in 2013 unless otherwise stated.

Table 1 shows a summary of socio-demographic characteristics of the cohort. The mean age was 30.0 years (SD 15.7) with more men than women. Most cases were from urban areas. The incidence of dengue increased towards the later part of the year (Fig 1).

**Table 1:** Socio-demographic characteristics of patients with dengue in Malaysia in 2013 (n = 43347).

Socio demographic characteristics	Total n (%)	Alive, n (%)	Dead, n (%), n = 92
**Age (n = 43347)**			
Mean (SD) (years)	30.0 (15.7)	30.0 (15.7)	41.8 (20.5)
Median (years)	28.0	28.0	42.5
Range (years)	<1–96	<1–96	<1–84
**Gender (n = 43347)**			
Male	26086 (60.2)	26042 (60.2)	42 (45.7)
Female	17261 (39.8)	17209 (39.8)	50 (54.3)
**Nationality (n = 43347)**			
Malaysian	29665 (91.5)	39574 (91.5)	87 (94.6)
Non Malaysian	3682 (8.5)	3677 (8.5)	5 (5.4)
**Ethnicity (n = 39662)**			
Malay	23486 (54.2)	23433 (59.2)	50 (57.5)
Chinese	10045 (23.2)	10021 (25.3)	24 (27.6)
Indian	4223 (9.7)	4216 (10.7)	6 (6.9)
Indigenous West Malaysia	172 (0.4)	172 (0.4)	0 (0.0)
Indigenous Sarawak	920 (2.1)	917 (2.3)	3 (3.4)
Indigenous Sabah	567 (1.3)	563 (1.4)	4 (4.6)
Others	249 (0.6)	249 (0.6)	0 (0.0)
**Place of dwelling (n = 38084)**			
Urban	32061 (84.2)	31990 (84.2)	68 (80.0)
Rural	6023 (15.8)	6005 (15.8)	17 (20.0)

Table 2 summarizes clinical manifestations at notification. The five most common accompanying features were body or joint ache, headache, nausea and/or vomiting, abdominal symptoms and rash.

**Table 2:** Clinical manifestations at notification (n = 43347).

Clinical manifestation	Total n (%)	Alive n (%), n = 43255	Dead, n (%), n = 92
Body or joint ache	37209 (85.8)	37134 (85.8)	73 (79.3)
Headache	32913 (75.9)	32849 (75.9)	63 (68.5)
Nausea and/or vomiting	14917 (34.4)	14867 (34.4)	47 (51.1)
Abdominal symptoms	12489 (28.8)	12455 (28.8)	31 (33.7)
Rash	6700 (15.5)	6689 (15.5)	11 (12.0)
Suborbital pain	1655 (3.8)	1653 (3.8)	2 (2.2)
Bleeding	864 (2.0)	856 (2.0)	7 (7.6)
Abdominal pain or tenderness	805 (1.9)	801 (1.9)	3 (3.3)
Lethargy and/or restlessness	316 (0.7)	310 (0.7)	6 (6.5)
Persistent vomiting	64 (0.1)	62 (0.1)	2 (2.2)
Severe plasma leakage	27 (0.1)	25 (0.1)	2 (2.2)
Liver enlargement 2cm	5 (<0.1)	5 (<0.1))	0 (0.0)
Shock	4 (<0.1)	1 (<0.1)	3 (3.3)
Clinical fluid accumulation	2 (<0.1)	2 (<0.1)	0 (0.0)
Organ failure (kidney/liver/central nervous system/heart)	2 (<0.1)	1 (<0.1)	1 (1.1)
Severe bleeding	0 (0.0)	0 (0.0)	0 (0.0)

Serological testing was done in 69.3% (30020) of the cohort of which 13081 were positive, 1210 were negative, 39 were equivocal and 15690 had no documented results. Table 3 shows the univariate and multivariate analysis of possible factors of dengue-related mortality (n = 41) in the serologically positive population. Multivariate analysis in those with confirmed dengue shows that increasing age, persistent vomiting, bleeding and severe plasma leakage were associated with mortality.

**Table 3:** Univariate and multivariate analysis (n = 13081) of dengue mortality in serologically-confirmed cases.

Variables	Alive n (%)	Dengue-related death, n (%)	Univariate analysis, OR (95% CI)	p	Multivariate analysis, OR (95% CI)	p
**Mean Age, years (SD)**	30.7 (15.86)	37.6 (21.19)	1.03 (1.01–1.04)	0.006	1.03 (1.01–1.05)	0.002
**Gender**						
Male	7580 (99.7)	19 (0.3)	1		1	
Female	5458 (99.6)	22 (0.4)	1.61 (0.87–2.97)	0.130	1.49 (0.80–2.78)	0.214
**Nationality**						
Malaysian	12188 (99.7)	38 (0.3)	1			
Non Malaysian	850 (99.6)	3 (0.4)	1.13 (0.35–3.68)	0.836		
**Race (n = 39658)**				0.580		
Malay	6011 (99.7)	18 (0.3)	1			
Chinese	4015 (99.7)	13 (0.3)	48.38 x 10^5^	0.997		
Indian	1241 (99.8)	2 (0.2)	52.30 x 10^5^	0.997		
Indigenous people	848 (99.4)	5 (0.6)	26.03 x 10^5^	0.998		
Others	71 (100.0)	0 (0.0)	95.25 x 10^5^	0.997		
**Place of dwelling (n = 38080)**						
Urban	9642 (99.7)	32 (0.3)	1			
Rural	1920 (99.6)	7 (0.4)	0.91 (0.40–2.07)	0.822		
**Month of onset**				0.918		
December 2012	166 (99.4)	1 (0.6)	1			
January	692 (99.4)	4 (0.6)	0.96 (0.11–8.64)	0.971		
February	593 (99.5)	3 (0.5)	0.84 (0.09–8.13)	0.880		
March	647 (99.7)	2 (0.3)	0.51 (0.05–5.69)	0.587		
April	595 (99.7)	2 (0.3)	0.56 (0.05–6.19)	0.635		
May	640 (99.7)	2 (0.3)	0.52 (0.05–5.76)	0.593		
June	945 (99.8)	2 (0.2)	0.35 (0.03–3.90)	0.394		
July	985 (99.9)	1 (0.1)	0.17 (0.01–2.71)	0.209		
August	1008 (99.5)	5 (0.5)	0.82 (0.10–7.09)	0.860		
September	1188 (99.7)	4 (0.3)	0.56 (0.06–5.03)	0.604		
October	1700 (99.7)	5 (0.3)	0.49 (0.06–4.20)	0.514		
November	2080 (99.8)	4 (0.2)	0.32 (0.04–2.87)	0.308		
December	1799 (99.7)	6 (0.3)	0.55 (0.07–4.63)	0.585		
**Headache**						
YES	9937 (99.7)	28 (0.3)	1		1	
NO	3101 (99.6)	13 (0.4)	1.48 (0.77–2.88)	0.237	1.46 (0.73–2.93)	0.283
**Suborbital Pain**						
YES	637 (99.8)	1 (0.2)	1			
NO	12401 (99.7)	40 (0.3)	2.05 (0.28–14.97)	0.477		
**Body or Joint Ache**						
YES	11390 (99.7)	31 (0.3)	1		1	
NO	1648 (99.4)	10 (0.6)	2.23 (1.09–4.56)	0.028	1.95 (0.90–4.25)	0.091
**Nausea and/or vomiting**						
YES	4171 (99.6)	18 (0.4)	1.66 (0.90–3.09)	0.106	1.38 (0.72–2.67)	0.327
NO	8867 (99.7)	23 (0.3)	1		1	
**Rash**						
YES	1967 (99.6)	7 (0.4)	1.16 (0.51–2.62)	0.723		
NO	11071 (99.7)	34 (0.3)	1			
**Abdominal symptoms**						
YES	3848 (99.7)	11 (0.3)	0.87 (0.44–1.75)	0.707		
NO	9190 (99.7)	30 (0.3)	1			
**Bleeding**						
YES	254 (98.1)	5 (1.9)	6.99 (2.72–17.96)	<0.001	5.84 (2.17–15.70)	<0.001
NO	12784 (99.7)	36 (0.3)	1		1	
**Abdominal pain or tenderness**						
YES	245 (99.2)	2 (0.8)	2.68 (0.64–11.15)	0.176	1.06 (0.19–5.92)	0.946
NO	12793 (99.7)	39 (0.3)	1		1	
**Persistent vomiting**						
YES	23 (92.0)	2 (8.0)	29.02 (6.61–127.31)	<0.001	13.34 (1.92–92.95)	0.009
NO	13015 (99.7)	39 (0.3)	1		1	
**Clinical fluid accumulation**						
YES	2 (100.0)	0 (0.0)				
NO	13036 (99.7)	42 (0.3)		1.00		
**Lethargy and/or restlessness**						
YES	108 (98.2)	2 (1.8)	6.14 (1.46–25.75)	0.013	0.94 (0.10–8.65)	0.955
NO	12930 (99.7)	39 (0.3)	1		1	
**Liver enlargement 2cm**						
YES	3 (100.0)	0 (0.0)				
NO	13035 (99.7)	41 (0.3)		0.999		
**Severe plasma leakage**						
YES	8 (80.0)	2 (20.0)	83.53 (17.19–405.94)	<0.001	66.68 (9.13–487.23)	<0.001
NO	13030 (99.7)	39 (0.3)	1		1	
**Shock**						
YES	0 (0.0)	3 (100.0)		0.999		
NO	13038 (99.7)	38 (0.3)				
**Severe bleeding**						
YES	0 (0.0)	0 (0.0)				
NO	13038 (99.7)	41 (0.3)				
**Organ failure**						
YES	0 (0.0)	1 (100.0)		0.999		
NO	13038 (99.7)	40 (0.3)				

Table 4 shows the univariate and multivariate analysis of possible factors of probably dengue-related mortality in the overall study population. Multivariate analysis shows that increasing age, female gender, nausea and/or vomiting, bleeding, lethargy and/or restlessness, severe plasma leakage and shock were associated with mortality.

**Table 4:** Univariate and multivariate analysis (n = 43343) of probable dengue-related mortality.

Variables	Alive n (%)	Dengue-related death, n (%)	Univariate analysis, OR (95% CI)	p	Multivariate analysis, OR (95% CI)	p
**Mean Age, years (SD)**	30.0 (15.7)	41.8 (20.5)	1.04 (1.03–1.05)	<0.001	1.04 (1.03–1.06)	<0.001
**Gender**						
Male	26042 (99.8)	42 (0.2)	1		1	
Female	17209 (99.7)	50 (0.3)	1.80 (1.20–2.72)	0.005	1.53 (1.01–2.33)	0.047
**Nationality**						
Malaysian	39574 (99.8)	87 (0.2)	1.62 (0.66–3.98)			
Non Malaysian	3677 (99.9)	5 (0.1)	1	0.297		
**Race (n = 39658)**				0.369		
Malay	23433 (99.8)	50 (0.2)	1			
Chinese	10021 (99.8)	24 (0.2)	1.12 (0.69–1.83)	0.642		
Indian	4216 (99.9)	6 (0.1)	0.67 (0.29–1.56)	0.349		
Indigenous people	1652 (99.6)	7 (0.4)	1.99 (0.90–4.39)	0.090		
Others	249 (100.0)	0 (0.0)	0.00 (0.00)	0.995		
**Place of dwelling (n = 38080)**						
Urban	31990 (99.8)	68 (0.2)	1			
Rural	6005 (99.7)	17 (0.3)	1.33 (0.78–2.27)	0.291		
**Month of onset**				0.752		
December 2012	486 (99.8)	1 (0.2)	1			
January	2283 (99.8)	5 (0.2)	1.30 (0.38–4.51)	0.675		
February	1751 (99.7)	5 (0.3)	0.68 (0.16–2.87)	0.605		
March	2000 (99.9)	3 (0.1)	0.71 (0.17–2.96)	0.635		
April	1938 (99.8)	3 (0.2)	0.82 (0.22–3.05)	0.766		
May	2230 (99.8)	4 (0.2)	1.16 (0.37–3.67)	0.796		
June	2747 (99.7)	7 (0.3)	0.65 (0.18–2.44)	0.528		
July	2789 (99.9)	4 (0.1)	1.10 (0.36–3.38)	0.862		
August	3309 (99.8)	8 (0.2)	1.71 (0.63–4.65)	0.291		
September	4532 (99.6)	17 (0.4)	0.85 (0.30–2.41)	0.759		
October	7095 (99.8)	12 (0.2)	0.77 (0.27–2.19)	0.628		
November	7095 (99.8)	12 (0.2)	0.89 (0.31–2.57)	0.830		
December	5639 (99.8)	11 (0.2)	0.94 (0.11–8.06)	0.955		
**Headache**						
YES	32489 (99.8)	63 (0.2)	0.69 (0.44–1.07)	0.688		
NO	10402 (99.7)	29 (0.3)	1			
**Suborbital Pain**						
YES	1653 (99.9)	2 (0.1)	1			
NO	41598 (99.8)	90 (0.2)	1.79 (0.44–7.27)	0.417		
**Body or Joint Ache**						
YES	37134 (99.8)	73 (0.2)	1		1	
NO	6117 (99.7)	19 (0.3)	1.58 (0.95–2.62)	0.076	1.52 (0.90–2.58)	0.120
**Nausea and/or vomiting**						
YES	14867 (99.7)	47 (0.3)	1.99 (1.32–3.00)	0.001	1.80 (1.17–2.77)	0.008
NO	28384 (99.8)	45 (0.2)	1		1	
**Rash**						
YES	6689 (99.8)	11 (0.2)	1			
NO	36562 (99.8)	81 (0.2)	1.35 (0.72–2.53)	0.354		
**Abdominal symptoms**						
YES	12455 (99.8)	31 (0.2)	1.26 (0.82–1.93)	0.301		
NO	30796 (99.8)	61 (0.2)	1			
**Bleeding**						
YES	856 (99.2)	7 (0.8)	4.08 (1.88–8.84)	<0.001	3.01 (1.29–7.04)	0.011
NO	42395 (99.8)	85 (0.2)	1		1	
**Abdominal pain or tenderness**						
YES	801 (99.6)	3 (0.4)	1.79 (0.56–5.66)	0.324		
NO	42450 (99.8)	89 (0.2)	1			
**Persistent vomiting**						
YES	62 (96.9)	2 (3.1)	15.48 (3.73–64.25)	<0.001	0.41 (0.01–18.35)	0.648
NO	43189 (99.8)	90 (0.2)	1		1	
**Clinical fluid accumulation**						
YES	2 (100.0)	0 (0.0)				
NO	43249 (99.8)	92 (0.2)		1.00		
**Lethargy and/or restlessness**						
YES	310 (98.1)	6 (1.9)	9.66 (4.19–22.28)	<0.001	5.97 (2.26–15.78)	<0.001
NO	42491 (99.8)	86 (0.2)	1		1	
**Liver enlargement 2cm**						
YES	5 (100.0)	0 (0.0)				
NO	43246 (99.8)	92 (0.2)		0.999		
**Severe plasma leakage**						
YES	25 (92.6)	2 (7.4)	38.42 (8.97–164.63)	<0.001	14.72 (1.54–140.67)	0.020
NO	43226 (99.8)	90 (0.2)	1		1	
**Shock**						
YES	1 (25.0)	3 (75.0)	1457.87 (150.21–14149.51)	<0.001	1805.37 (125.44–25982.98)	<0.001
NO	43250 (99.8)	89 (0.2)	1		1	
**Severe bleeding**						
YES	0 (0.0)	0 (0.0)				
NO	43251 (99.8)	92 (0.2)				
**Organ failure**						
YES	1 (50.0)	1 (50.0)	475.28 (29.50–7656.58)	<0.001	2.947 (0.00–9.74x10^10^)	0.923
NO	43250 (99.8)	91 (0.2)	1		1	

Table 5 shows the univariate and multivariate analysis of possible factors of dengue-related mortality in the adult study population (age 15 years and above). Multivariate analysis shows that increasing age, nausea and/or vomiting, lethargy or restlessness, and severe plasma leakage were associated with mortality.

**Table 5:** Univariate and multivariate analysis (n = 37021) of probable dengue-related mortality in Adults (≥15 years old).

Variables	Alive n (%)	Dengue-related death, n (%)	Univariate analysis, OR (95% CI)	p	Multivariate analysis, OR (95% CI)	p
**Mean Age, years (SD)**	33.6 (13.95)	45.2 (18.05)	1.05 (1.04–1.06)	<0.001	1.05 (1.04–1.06)	<0.001
**Gender (n = 37018)**						
Male	22478 (99.8)	40 (0.2)	1		1	
Female	14456 (99.7)	44 (0.3)	1.71 (1.11–2.63)	0.014	1.34 (0.87–2.08)	0.190
**Nationality**						
Malaysian	33352 (99.8)	79 (0.2)	1.70 (0.69–4.19)	0.252		
Non Malaysian	3582 (99.9)	5 (0.1)	1			
**Race (n = 33427)**				0.622		
Malay	19372 (99.8)	45 (0.2)	1			
Chinese	8778 (99.8)	22 (0.3)	1.08 (0.65–1.80)	0.771		
Indian	3539 (99.8)	6 (0.2)	073 (0.31–1.71)	0.469		
Indigenous people	1439 (99.6)	6 (0.4)	1.80 (0.77–4.21)	0.179		
Others	220 (100.0)	0 (0.0)	0	0.996		
**Place of dwelling (n = 32570)**						
Urban	27304 (99.8)	63 (0.2)	1			
Rural	5189 (99.7)	14 (0.3)	1.17 (0.66–2.09)	0.597		
**Month of onset**				0.660		
December 2012	407 (99.8)	1 (0.2)	1			
January	1938 (99.8)	4 (0.2)	0.84 (0.09–7.54)	0.876		
February	1477 (99.7)	5 (0.3)	1.38 (0.16–11.83)	0.770		
March	1693 (99.9)	2 (0.1)	0.48 (0.04–5.32)	0.550		
April	1644 (99.8)	3 (0.2)	0.74 (0.08–7.16)	0.797		
May	1933 (99.9)	2 (0.1)	0.42 (0.04–4.66)	0.481		
June	2396 (99.8)	6 (0.2)	1.02 (0.12–8.49)	0.986		
July	2394 (99.8)	4 (0.2)	0.68 (0.08–6.10)	0.730		
August	2867 (99.8)	7 (0.2)	0.99 (0.12–8.10)	0.995		
September	3835 (99.6)	16 (0.4)	1.79 (0.23–12.84)	0.608		
October	5431 (99.8)	12 (0.2)	0.90 (0.12–6.93)	0.919		
November	6114 (99.8)	12 (0.2)	0.80 (0.10–6.16)	0.829		
December	4805 (99.8)	10 (0.2)	0.85 (0.11–6.63)	0.874		
**Headache**						
YES	28606 (99.8)	60 (0.2)	1			
NO	8328 (99.7)	24 (0.3)	1.37 (0.86–2.21)	0.189		
**Suborbital pain**						
YES	1477 (99.9)	2 (0.1)	1			
NO	35457 (99.8)	82 (0.2)	1.71 (0.42–6.95)	0.455		
**Body or joint ache**						
YES	32353 (99.8)	70 (0.2)	1		1	
NO	4581 (99.7)	14 (0.3)	1.41 (0.80–2.51)	0.239	1.26 (0.70–2.27)	0.451
**Nausea and/or vomiting**						
YES	12422 (99.7)	42 (0.3)	1.97 (1.229–3.03)	0.002	1.90 (1.22–2.98)	0.005
NO	24512 (99.8)	42 (0.2)	1		1	
**Rash**						
YES	5449 (99.8)	9 (0.2)	1			
NO	31485 (99.8)	75 (0.2)	1.44 (0.77–2.88)	0.300		
**Abdominal symptoms**						
YES	10493 (99.7)	27 (0.3)	1.19 (0.76–1.89)	0.449		
NO	26441 (99.8)	57 (0.2)	1			
**Bleeding**						
YES	750 (99.5)	4 (0.5)	2.41 (0.88–6.60)	0.086	1.95 (0.68–5.59)	0.213
NO	26184 (99.8)	80 (0.2)	1		1	
**Abdominal pain or tenderness**						
YES	670 (99.6)	3 (0.4)	2.01 (0.63–6.36)	0.238	1.05 (0.28–3.98)	0.943
NO	36264 (99.8)	81 (0.2)	1		1	
**Persistent vomiting**						
YES	50 (96.2)	2 (3.8)	17.99 (4.31–75.17)	<0.001	2.99 (0.34–26.38)	0.325
NO	36884 (99.8)	82 (0.2)	1		1	
**Clinical fluid accumulation**						
YES	2 (100.0)	0 (0.0)				
NO	36932 (99.8)	84 (0.2)		1.00		
**Lethargy and/or restlessness**						
YES	251 (97.7)	6 (2.3)	11.24 (4.86–26.03)	<0.001	5.98 (2.13–16.81)	0.001
NO	36683 (99.8)	78 (0.2)	1		1	
**Liver enlargement 2cm**						
YES	2 (100.0)	0 (0.0)				
NO	36932 (99.8)	84 (0.2)		1.00		
**Severe plasma leakage**						
YES	17 (89.5)	2 (10.5)	52.97 (12.04–232.93)	<0.001	23.32 (2.58–229.10)	0.005
NO	36917 (99.8)	82 (0.2)	1		1	
**Shock**						
YES	1 (50.0)	1 (50.0)	444.98 (27.60–7173.74)	<0.001	2.19 (0.00–22954.64)	0.868
NO	36933 (99.8)	83 (0.2)	1		1	
**Severe bleeding**						
YES	0 (0.0)	0 (0.0)				
NO	36934 (99.8)	84 (0.2)				
**Organ failure**						
YES	1 (50.0)	1 (50.0)	444.98 (27.60–7173.74)	<0.001	27.72 (0.00–235825.80)	0.472
NO	36933 (99.8)	83 (0.2)	1		1	

## Discussion

The large cohort is a reflection of the increase in the incidence of dengue in the past decade [15]. The case fatality rate of 2 in a thousand is consistent with that found over the years since 2001 [6, 7]. With proper identification of factors associated with dengue mortality, time and resources can be focused on those at highest risk.

Our findings show that among those who were serologically confirmed, increasing age, persistent vomiting, bleeding and severe plasma leakage were independently associated with mortality. This differed from the overall study population because it included persistent vomiting, and excluded female gender, nausea and/or vomiting, lethargy and/or restlessness and shock. These added factors are non-specific and one possible explanation is that the death could have been due to other non-dengue illnesses. However, there were only 41 deaths in the serologically confirmed group which represented 44.6% of overall deaths. Only 30.2% of the entire study population (13081 of 43343) were serologically positive. The relatively small number of deaths which is the outcome of interest limits the prognostic information available even with the large study population. We included data from the entire study population as the diagnosis was made based on the WHO clinical criteria for dengue. For those with serological results, 91.3% were positive showing that clinical diagnosis is highly predictive of dengue. Determination of early warning symptoms and signs in the overall population regardless of serological confirmation is crucial in actual clinical practice as this is the population that presents to primary care. The burden of caring for those who are clinically suspected to have dengue is overwhelming as many of the early symptoms and signs are non-specific. Yet, dengue is endemic and is potentially fatal. Early warning symptoms and signs can help the primary care practitioner to identify those who are at greater risk of complications even when diagnostic test results are not available.

We found that increasing age was associated with mortality in the serologically confirmed as well as the overall study cohort. The mean age of those who died from dengue was 12 years greater than those who survived. The number of children who died from dengue in this study population was very small and we were unable to perform a multivariate analysis on this sub-group. In the multivariate analysis of the adult subgroup, increasing age remained a significant associated factor. Older age was found to be associated with severe dengue in France [11]. This differed from a study in Vietnam which found that mortality was highest in young children [12]. A meta-analysis by Huy et al found that age was negatively associated with dengue shock syndrome. However, there was high heterogeneity and this association was mainly seen in studies on children. They were unable to analyse the age factor in adults [13]. The number of children who died from dengue in this study population was very small and we were unable to perform any analysis in this sub-group.

Although gender was associated with mortality in the overall study population, this was not seen in the subgroup analysis for adult nor the serologically confirmed group. Studies have conflicting evidence on the association of gender with severe dengue or dengue-related death. A meta-analysis, and studies in Singapore and Vietnam found that female gender was associated with severe dengue [12, 13, 16]. However, a study in France showed that male gender was associated with severe dengue manifestations [11]. It is unclear as to the mechanism by which age and gender affects the disease manifestation and outcomes. Studies have reported that gender differences could be due to differences in physiology [17] or health seeking behavior [13, 16].

This study found that persistent vomiting, bleeding and severe plasma leakage was associated with dengue-related mortality in the serologically confirmed group while nausea and/or vomiting, bleeding, lethargy, severe plasma leakage and shock were associated with dengue-related mortality in the overall population. Of these, only nausea and/or vomiting has not been included as a warning sign in guideline recommendations [6]. Nausea and/or vomiting should be considered as an early warning sign that can alert the attending health care practitioner to monitor such patients closely. Nausea and vomiting were also found to be associated with dengue shock syndrome in a meta-analysis and other studies [13, 18]. These symptoms could lead to reduced fluid intake and hence dehydration and serious complications during the phase of plasma leakage. Primary care practitioners should be alerted to these warning signs, as they are the first line contacts for these patients at risk. Bleeding, shock and severe plasma leakage are late manifestations. It is important to detect bleeding and plasma leakage early by monitoring hematocrit levels frequently. Frequent testing during the critical period will guide clinicians in adjusting the rate of intravenous fluid replacement to prevent severe plasma leakage and therefore avert shock. Thrombocytopenia is also useful in detecting early plasma leakage and bleeding. Thus, a complete blood count with haematocrit, white cell and platelet counts is a simple laboratory test that is worth doing to detect and avert these complications [19].

As our data were obtained at notification, some patients presented late in the disease process. Hence the public needs to be educated about dengue and its presentation so that they seek treatment earlier.

We found that the incidence of dengue was greater in the later part of the year. There is evidence that dengue incidence is associated with climatic factors, such as temperature and rainfall [15, 20]. A study in Malaysia showed that rainfall peaked in April and through October to December [21].

### Strengths and limitation

The large database of more than 40 000 individuals with significant numbers of mortality enabled the identification of its independent associated factors.

In the process of data cleaning, we found that important factors such as blood indices (platelet count, haematocrit and white cell count) could not be used due to data input error. For example, platelet count were entered as 1.39, 139 or 139 000. In order to ensure that the data were clean, we omitted those variables that could not be verified. Hospitalisation was not captured as a variable in the database and therefore could not be used as a study outcome.

### Recommendation

We recommend that further training be given to those who enter the data. A glossary of variables for the database should be developed. In addition, data that is entered needs to be verified by a medical personnel before final entry.

For clinical practice, doctors treating dengue especially those working in the front-line should be made aware of the independent associated factors of dengue mortality. Future studies should include death cases from other years as different serotypes may predominate in different years.

### Conclusion

The case fatality rate remains at 2 in a thousand in this cohort. Older persons and patients with persistent vomiting, bleeding or severe plasma leakage, which were associated with mortality, at notification should be monitored closely and referred early for hospitalisation.
